# Changes in knowledge of safe sex and sexual behaviour among male vocational high school students in Zhejiang Province, China: a 6-year cross-sectional comparison (2015–2021)

**DOI:** 10.3389/frph.2025.1653622

**Published:** 2025-09-24

**Authors:** Hui Wang, Qiaoqin Ma, Lin He, Tingting Jiang, Wanjun Chen, Jinglei Zhen, Weiyong Chen

**Affiliations:** Department of HIV/STD Prevention and Control, Zhejaing Provincial Center for Disease Prevention and Control, Hangzhou, China

**Keywords:** vocational high school, male students, safe sex knowledge, sexual behaviour, change

## Abstract

**Background:**

Vocational high school students in China exhibit higher rates of sexual activity than their academic counterparts; however, existing studies predominantly focus on college populations, leaving a critical gap in longitudinal data tracking the evolution of sexual knowledge and behavioural patterns among vocational students. This study aimed to evaluate changes over time in sexual safety knowledge, STIs/HIV and reproductive health-related sexual behaviours among boys who have had sex in Chinese vocational high schools.

**Methods:**

In 2015 and 2021, an anonymous survey was conducted separately using the same questionnaire and survey method among students in Years 1–2 of the same vocational high school in a district of Zhejiang Province. The results of the two surveys were compared using univariate analyses, and multivariate analyses.

**Results:**

In 2015 and 2021, 180 and 90 male students, respectively, had ever had sex, representing rates of 17.44% (180/1,032) and 8.49%(90/1,060). The results of the multivariate analysis showed that, in 2021, those who knew that sexual contact was the main mode of HIV transmission(OR = 14.14, 95% CI: 7.16–27.92), knew that contracting an STI increased the likelihood of HIV infection (OR = 7.83, 95% CI: 4.30–14.29), and knew that condom use could reduce both STIs(OR = 33.51, 95% CI: 15.08–74.49) and HIV infection(OR = 16.77, 95% CI: 8.58–32.76) were significantly more prevalent than in 2015. Furthermore, students who had received sex and STI/HIV prevention education and rated it as "good" (OR = 5.62, 95% CI: 2.72–11.64), who believed they could confidently refuse sex without a condom(OR = 1.95, 95% CI: 1.14–3.32), and who reported consistent condom use(OR = 2.27, 95% CI: 1.21–4.25) were also more prevalent in 2021. Use of contraception methods such as condoms(OR = 2.76, 95% CI: 1.50–5.09), the pill(OR = 2.42, 95% CI: 1.35–4.35), extracorporeal ejaculation (OR = 1.77, 95% CI:1.02–3.09), and the safe period(OR = 2.22, 95% CI: 1.22–4.04) also increased significantly. However, the proportion of students who had their first sexual relationship with a non-regular partner decreased(OR = 0.47, 95% CI: 0.24–0.93).

**Conclusion:**

The participants' knowledge of safe sex and sexual behaviour improveed between the two surveys. However, these positive changes have not had a significant enough impact to prevent STIs/HIV transmission and reduce pregnancy. Further efforts are needed to ensure that these changes lead to effective STIs/HIV prevention and reduction among students.

## Introduction

Adolescence is a unique period during which young people develop behavioural patterns that can either protect their own and others' health, or expose them to present and future health risks (1). Adolescent sexual health and sexually transmitted infections (STIs) are major global public health concerns. Adolescents' sexual attitudes, behaviours and health risks are evolving, their sexual and reproductive health needs are more pronounced than those of adults because they are more likely to have multiple concurrent sexual partners, and are less likely to use condoms than other age groups. They are also more physiologically vulnerable due to cervical immaturity, and less likely to seek healthcare than adults. In general, STIs are on the rise among adolescents worldwide, although HIV infection and unintended pregnancy among adolescents are declining, they still require great attention (1, 2).

In recent years, the sexual attitudes and behaviours of Chinese adolescents have changed significantly due to the acceleration of socio-economic development and information dissemination (3). China's HIV epidemic surveillance data shows that the HIV epidemic has spread to schools, with an increasing number of cases among high school students (4). The incidence of STIs, including HIV infection, has also increased significantly among adolescents (5). Students at vocational high school (VHS) face higher sexual health risks due to their specific educational background and social environment, and they engage in sexual behaviours at significantly higher rates than regular high school (RHS) students (6–10). For instance, a survey in Zhejiang Province revealed that 10.8% of VHS students had engaged in sexual behaviour, compared to 5.8% of RHS students (8). VHS students are more likely to have non-regular sexual partners, and to have had more sexual partners, which increases their risk of contracting STI and HIV infection (11). In addition, unplanned pregnancies and abortions among VHS students are also worrying, with about 3/4 of abortions among high school students being performed by VHS students (11).

VHS students receive relatively little sexual health education (6, 12), and a significantly lower proportion receive sex education from their parents than RHS students (12). VHS students also differ from RHS students in terms of their sexual knowledge and awareness of safe sex practices. Studies have shown that VHS students have lower levels of knowledge regarding HIV transmission (7, 9, 10, 12), reproductive health (10, 12) and STI and HIV prevention (6) than RHS students. VHS students viewed more pornographic material and were more likely to have positive attitudes towards high school students' sexual behaviour than RHS students (7, 10). This knowledge gap may lead VHS students to be less aware of the consequences of sexual behaviour and to take fewer protective measures, thereby increasing their risk of contracting STIs/HIV and unwanted pregnancy.

A through understanding of VHS students' sexual behaviour, knowledge and sex education needs is crucial for developing targeted interventions to prevent the spread of STIs/HIV and unwanted pregnancies. This study aims to identify changes in VHS students' sexual behaviour and knowledge, and to evaluate the effectiveness of sex and STIs/HIV prevention education in recent years. This will be achieved by comparing and analysing data from a study conducted among VHS students in Zhejiang Province in 2015 and 2021.

## Methods

### Study design, location and participants

A cross-sectional survey was conducted in a VHS school in the central region of Zhejiang Province, China, in 2015 and again in 2021 using the same questionnaire. Zhejiang Province, located in the eastern coastal area of China, is a developed region that has experienced rapid economic growth, with its per capita gross domestic product (GDP) increasing from RMB 68,569 in 2014 to RMB 125,043 in 2023 (13).

The inclusion criteria for the study participants in the 2015 and 2021 surveys were as follows: 1. Students in Years 1 and 2 who were studying at the survey school at the time of the survey; 2. Students and their parents who agreed to participate in the survey; 3. Students who did not have a mental health condition or other health problem that would prevent them from completing the questionnaire independently.

### Sample size and sampling

Sample size estimations were based on the proprtions of sexual behaviour reported in the recent literature among VHS students in Zhejiang Province prior to the 2015 and 2021 surveys, using the formual *n* = Z^2^*α* × p(1-p)/d^2^, where Z*α* = 1.96, *α* = 0.05, *p* = reported proportion of sexual behavior, d = *p* × 0.15.

In 2013, the proportion of VHS students engaging in sexual behaviour was reported to be 10.81% (8). At least 1,468 subjects had to be recruited for the 2015 survey. In 2018, the proportion was reported to be 13.05% (9); at least 1,185 subjects needed to be surveyed in 2021.

In 2015, 2,196 Years 1–2 students were enrolled in the surveyed school, with 1,839 surveyed (1,032 males). In 2021, 2,051 students in both Years were enrolled, with 1,960 surveyed (1,060 males). Sexual behaviour was reported by 266(180 males) in 2015 and 128 (90 males) in 2021. This paper analyses the male students who have had sex.

### Study procedure

Both the 2015 and 2021 surveys employed the same questionnaire and research methods in the same school. Both surveys were conducted in classrooms in April and May of their respective years. The investigators were teachers from the surveyed school and students from a local university. The research team provided the investigators with uniform training, covering the purpose of the survey, its methodology, and its requirements. The survey was aimed at all students in Years 1 and 2 at the school.

Prior to commencing the survey, the investigator explained its purpose, methodology and privacy policy to the students' guardians via the parents' WeChat group. This included information on how the questionnaire data would be used. It was made clear that only aggregated statistical data would be used, not personal data, and that no information from the students' questionnaires would be disclosed to school teachers, administrators or other students. Non-participation would not result in any disadvantages. This information was also provided to the students in class and included at the beginning of the questionnaire. After school, the students took the informed consent form home. After careful reading, it was signed by their guardians. During the formal survey, students sat one seat apart from each other. Having read the informed consent section at the beginning of the questionnaire, students indicated their agreement by ticking a box. Once completed, the questionnaire was collected by the investigator. The survey protocols were reviewed and approved by the Ethical Review Committee of the Zhejiang Provincial Centre for Disease Control and Prevention.

### Study variables

In 2015, the research team developed a preliminary questionnaire based on a review of domestic and international literature (8, 14–16). This was continuously revised and improved through in-depth individual and group interviews with students from a VHS outside the study VHS and repeated discussions within the research team. The final questionnaire consisted of six sections covering general demographic characteristics, lifestyle and self-assessment, sexual safety knowledge, sexual attitudes, sexual behaviour and risk awareness, safer sex self-efficacy, exposure to sex education and STIs/HIV prevention education at school and assessment. The number of questions in each section was 9, 13, 13, 6, 16 and 5 respectively.

Engineering disciplines in the text include computer applications, network technology, electrical and electronic engineering, numerical control engineering, mould design and fabrication, electrical equipment maintenance and mechatronics; liberal arts disciplines include foreign trade marketing, finance and accounting, preschool education, and art and decoration.

A nuclear family consists of parents and their unmarried children, whereas an extended family consists of parents and their unmarried children, as well as other related family members. Being sexually active means that one of the sexual acts of vaginal, oral or anal sex has taken place.

### Data analysis

Data from the two surveys in 2015 and 2021 were combined and statistically analysed using SPSS 23.0 statistical software. The *χ*^2^ test was used to compare statistical differences between the two surveys in the distribution of demographic characteristics, knowledge of safe sex, self-efficacy for safe sex, exposure to school sexual safety education, and sexual behaviour-related variables among students who had already had sex.

Variables with a *P* value <0.05 in the univariate analyses were used as dependent variables, and demographic variables such as year of survey, Year, major, family type, father's literacy, mother's literacy, family economic status and residence were included as independent variables in the corresponding multivariate logistic regression models. The variable for school stage of sexual initiation was also included in the logistic regression model for sexual behaviour-related variables to adjust for possible confounding. Odds ratios (OR) and 95% confidence intervals (CI) were used to describe the relationship between year of survey and the corresponding variables. A *P* value of <0.05 was used to indicate a significant difference.

## Resluts

### Characteristics of demographic change between the two surveys

The proportion of male students who were sexually active decreased from 17.44% (180/1,032) in 2015 to 8.49% (90/1,060) in 2021. Among thosee who were sexually active, approximately 47% were first-year students in both years (Table 1). The proportion of engineering majors dropped from 59.4% to 51.1%, while the proportion of students from nuclear families decreased from 76.1% to 64.4%. The proportion of fathers with a junior high school education or less fell from 64.4% to 48.9%, while the proportion for mother decreased from 74.4% to 58.9%. Most students(84%–87%) came from average or higher economic families, and most (71.1% –73.3%) lived at home.

**Table 1:** Demographic characteristics of the sexually active boys between two surveys.

Variables	2015 (*n* = 180)	2021 (*n* = 90)	*χ*^2^ value	*P* value
Year				
One	85 (47.2)	42 (46.7)	0.007	0.931
Two	95 (52.8)	48 (53.3)		
Major				
Engineering	107 (59.4)	46 (51.1)	1.697	0.193
Liberal arts	73 (40.6)	44 (48.9)		
Family type				
Nuclear family	137 (76.1)	58 (64.4)	4.315	0.116
Extended family	33 (18.3)	26 (28.9)		
The other	10 (5.6)	6 (6.7)		
Fathers' literacy				
Junior high school/below	116 (64.4)	44 (48.9)	6.014	0.014
High school/above	64 (35.6)	46 (51.1)		
Mothers' literacy				
Junior high school/below	134 (74.4)	53 (58.9)	6.819	0.009
High school/above	46 (25.6)	37 (41.1)		
Family's economic status				
Average/rich	158 (87.8)	76 (84.4)	0.577	0.448
Poor	22 (12.2)	14 (15.6)		
Residence				
School dormitory	43 (23.9)	21 (23.3)	0.424	0.809
Home	128 (71.1)	66 (73.3)		
The other	9(5.0)	3(3.3)		

The *χ*² tests indicated significant differences in parents' literacy levels (*P* = 0.014 for fathers, and *P* = 0.009 for mothers).

### Characteristics of changes in safe sex knowledge, safe sex self-efficacy, and exposure to and evaluation of school-based education between the two surveys

Among sexually active boys, the proportion of students with key HIV/STI-related knowledge and positive attitudes increased significantly in 2021 compared to 2015 (all *P* < 0.05; Table 2): knowing that the main mode of HIV transmission is sexual contact rose from 27.8% in 2015 to 84.4% in 2021; understanding that contracting an STI increases the likelihood of HIV infection grew from 27.2%–72.2%; knowledge of condom use reducing STIs increased from 21.7%–90.0%; knowledge of condom use reducing HIV infection rose from 21.1%–81.1%; confidence in refusing sex without a condom increased from 43.9%–58.9%; and the percentage having received school-based sex and STI/HIV prevention education and rated it as “good” surged from 19.1%–55.0%.

**Table 2:** Characteristics of safe sex knowledge, safe sex self-efficacy and exposure to and evaluation of school-based education among sexually active boys between two surveys.

Variables	2015 (*n* = 180)	2021 (*n* = 90)	*χ*^2^ value	*P* value
The main mode of HIV transmission was sexual contact				
Incorrect/Unknown	130 (72.2)	14 (15.6)	77.411	<0.001
Correct	50 (27.8)	76 (84.4)		
Contracting STI were more likely to be infected with HIV				
Incorrect/Unknown	131 (72.8)	25 (27.8)	49.805	<0.001
Correct	49 (27.2)	65 (72.2)		
Condom use can reduce STIs				
Incorrect/Unknown	141 (78.3)	9 (10.0)	113.468	<0.001
Correct	39 (21.7)	81 (90.0)		
Condom use can reduce HIV infection				
Incorrect/Unknown	142 (78.9)	17 (18.9)	89.220	<0.001
Correct	38 (21.1)	73 (81.1)		
Confidently refuse to have sex without a condom				
Not confident/Unsure	101 (56.1)	37 (41.1)	5.403	0.020
Confident	79 (43.9)	53 (58.9)		
Exposure to sex education and STI/ HIV prevention education				
No	70 (38.9)	30 (33.3)	0.794	0.373
Yes	110 (61.1)	60 (66.7)		
Evaluation of sex education and STI/ HIV prevention education^a^				
Average/Need improvement	89 (80.9)	27 (45.0)	23.097	<0.001
Good	21(19.1)	33(55.0)		

^a^ The denominator is students who reported having been exposed to sex education and STI/ HIV prevention education, which was 110 in 2015 and 60 in 2021.

### Characteristics of changes in sexual behaviour between the two surveys

Significant changes were observed in the sexual behaviors of the students in 2021 compared to 2015 (all *P* < 0.05; Table 3): first sexual relationship with a non-regular partner decreased from 29.4% to 17.8%; oral sex engagement rose from 37.8%–58.6%; anal sex prevalance increased from 11.7%–26.1%; consistent condom use improved from 17.8%–35.2%; condoms use for contraception grew from 52.8%–74.7%; the use of other contraceptive methods such as the pill (from 25.0%–45.9%), extracorporeal ejaculation (from 35.6%–52.4%), and the safe period (from 25.0%–40.7%) also showed significant increase.

**Table 3:** Characteristics of sexual behaviour among sexually active boys between two surveys.

Variables	2015 (*n* = 180)	2021 (*n* = 90)^a^	*χ*^2^ value	*P* value
School stage of sexual initiation				
Junior high school/before	71 (39.4)	29 (34.1)	0.697	0.404
High school	109 (60.6)	56 (65.9)		
Partner type of first sex				
Regular	127 (70.6)	74 (82.2)	4.293	0.038
Non-regular	53 (29.4)	16 (17.8)		
Sex of sexual partner				
Female	166 (92.2)	79 (87.8)	1.411	0.235
Male/Both	14 (7.8)	11 (12.2)		
Ever oral sex				
No	112 (62.2)	36 (41.4)	10.314	0.001
Yes	68 (37.8)	51 (58.6)		
Ever anal sex				
No	159 (88.3)	65 (73.9)	9.018	0.003
Yes	21 (11.7)	23 (26.1)		
Number of sexual partner				
1	117 (65.0)	55 (63.2)	0.081	0.776
≥2	63 (35.0)	32 (36.8)		
Frequency of condom use				
Never/sometimes	148 (82.2)	57 (64.8)	10.008	0.002
Every time	32 (17.8)	31 (35.2)		
Ever using condoms for contraception				
No	85 (47.2)	22 (25.3)	11.751	0.001
Yes	95 (52.8)	65 (74.7)		
Ever using the pill for contraception				
No	135 (75.0)	46 (54.1)	11.629	0.001
Yes	45 (25.0)	39 (45.9)		
Ever using extracorporeal ejaculation for contraception				
No	116 (64.6)	40 (47.6)	7.707	0.010
Yes	64 (35.6)	44 (52.4)		
Ever using safe period contraception				
No	135 (75.0)	48 (59.3)	6.605	0.010
Yes	45 (25.0)	33 (40.7)		
Pregnancy of female sexual partner				
No	168 (93.3)	85 (97.7)	2.252	0.133
Yes	12 (6.7)	2 (2.3)		
STI diagnosis				
No	177 (98.3)	88(97.8)	0.102	0.750
Yes	3 (1.7)	2 (2.2)		

^a^ The subject may not add up to 90 due to missing data.

Chi-square tests reveale no significant differences between the two surveys for the following variables: school stage of sexual initiation, sex of sexual partner, number of sexual partners, experience of pregnancy with a female sexual partner, and experience of an STI diagnosis.

### Results of multivariate logistic regression analysis

A multivariate logistic regression model was established for each variable demonstrating a significant difference in distribution in the univariate analysis. After adjusting for demographic variables (year of survey, year, major, family type, father's literacy, mother's literacy, family economic status, and residence), the following factors exhibited markedly elevated odds ratios in 2021 compared to 2015 (Table 4): knowing that sexual contact is the main mode of HIV transmission(OR = 14.14, 95% CI:7.16–27.92), knowing that contracting an STI increases the likelihood of HIV infection (OR = 7.83, 95% CI: 4.30–14.29), knowing that condom use can reduce STIs (OR = 33.51, 95% CI: 15.08–74.49) and HIV infection (OR = 16.77, 95% CI: 8.58–32.76), believing that they would confidently refuse to have sex without a condom(OR = 1.95, 95% CI: 1.14–3.32), and having received and rated school sex education and STI/HIV prevention education as “good” (OR = 5.62, 95% CI: 2.72–11.64).

**Table 4:** Results of multivariate logistic analysis.

Variables	Adjusted OR(95% CI)^a^	*P* value
The main mode of HIV transmission was sexual contact		
Incorrect/Unknown	1	
Correct	14.14 (7.16–27.92)	<0.001
Contracting STI were more likely to be infected with HIV		
Incorrect/Unknown	1	
Correct	7.83 (4.30–14.29)	<0.001
Condom use can reduce STIs		
Incorrect/Unknown	1	
Correct	33.51 (15.08–74.49)	<0.001
Condom use can reduce HIV infection		
Incorrect/Unknown	1	
Correct	16.77 (8.58–32.76)	<0.001
Confidently refuse to have sex without a condom		
Not confident/Unsure	1	
Confident	1.95 (1.14–3.32)	<0.014
Evaluation of sex education and HIV/STI prevention education		
Average/Need improvement		
Good	5.62 (2.72–11.64)	<0.001
Partner type of first sex^a^		
Regular	1	
Non-regular	0.51 (0.27–0.98)	0.042
Ever oral sex^a^		
No	1	
Yes	2.60 (1.47–4.60)	0.001
Ever anal sex^a^		
No	1	
Yes	2.65 (1.28–5.48)	0.008
Frequency of condom use^a^		
Never/sometimes	1	
Consistent	2.27 (1.21–4.25)	0.010
Ever using condoms for contraception^a^		
No	1	
Yes	2.76 (1.50–5.09)	0.001
Ever using the pill for contraception^a^		
No	1	
Yes	2.42 (1.35–4.35)	0.003
Ever using extracorporeal ejaculation for contraception^a^		
No	1	
Yes	1.77 (1.02–3.09)	0.043
Ever using safe period contraception*		
No	1	
Yes	2.22 (1.22–4.04)	0.009

^a^ School stage of sexual initiation was further included in the multivariate logistic model to adjust for possible confounding.

Sexual behaviour variables demonstrating significant differences in the univariate analyses were further adjusted for school stage of sexual initiation in addition to demographic variables. The multivariate logistic regression analysis revealed that the following were significantly more prevalent in 2021 than in 2015: oral sex (OR = 2.60, 95% CI: 1.47–4.60) and anal sex (OR = 2.65, 95% CI: 1.28–5.48), consistent condom use(OR = 2.27, 95% CI: 1.21–4.25), and various contraceptive methods such as condoms use(OR = 2.76, 95% CI: 1.50–5.09), the pill (OR = 2.42, 95% CI: 1.35–4.35), extracorporeal ejaculation (OR = 1.77, 95% CI: 1.02–3.09), and the safe period (OR = 2.22, 95% CI: 1.22–4.04). However, the proportion of students who initiated sexual relationship with non-regular partners decreased (OR = 0.47, 95% CI: 0.24–0.93).

## Discussion

The present study was conducted using the same questionnaire and survey methodology among students in Years 1–2 in the same school, six years apart. The study revealed positive changes in the rates of sexual initiation, knowledge, and self-efficacy regarding sexual safety, as well as in various sexual behaviors among boys who had already had sex in VHS.

From 2015–2021, there was a notable increase in the knowledge of the study population regarding the main mode of HIV transmission being sexual contact and the effectiveness of condom use in reducing STIs and HIV infection, with adjusted ORs above 14. Additionally, the understanding that contracting STIs increases the likelihood of HIV infection also improved, with an adjusted OR of 7.8. There was a significant increase in the proportion of students who received and rated sexual health and STIs/HIV prevention education at school as good. Schools are the main place for adolescents to receive sex education and education on the prevention of STIs/HIV (17, 18), and comprehensive school-based education can significantly improve adolescents' knowledge of sexual and reproductive health and STIs/HIV prevention, as well as reducing risky sexual attitudes and behaviours (17–19). National surveillance data for male high school students in the United States showed a decrease in the percentage engaging in sexual intercourse, falling from 47.0% to 32.0% between 2013 and 2023 (20). The 2021 survey results were significantly lower than the aforementioned US surveillance data in 2023 (20), lower than 9.0% (8) and 10.5% (9) of male students in Years 1–3 at VHS in this province, 9.3% of male students in Years 1–3 at RHS in Guangdong province (21), and closer to 8.7% of male students at RHS in Guizhou Province (22). This study reported a significant decrease in the proportion of students engaging in sexual behaviour, suggesting that VHS students are making better choices in this regard. Students with higher levels of knowledge about sexual and reproductive health and disease prevention tend to initiate sexual behaviour later (18, 23). We report a significant improvement in parental literacy among male students in 2021, and higher parental literacy is associated with delayed onset of sexual behaviour in their children (24, 25). However, the overall low level of parental literacy in the study suggests that there is a need to help students' parents to understand the importance of sex education and to master the correct methods, and to encourage them to have open and healthy communication with their children. This finding is encouraging, given that the literature reports that an association between early sexual intercourse and multiple sexual partners, unprotected sex, unwanted pregnancy, STIs and HIV infection (26–28).

The proportion insisting on condom use differed significantly between the two surveys. This may be related to the higher level of knowledge about HIV/STIs among study participants in the second survey and their perception that condom use can prevent STIs/HIV. Chinese literature reports that college students with higher levels of sexual and reproductive health knowledge are less likely to engage in unprotected sex (18), and Thai VHS students with high levels of HIV knowledge exhibit higher rates of condom use (29). The two surveys were conducted 6 years apart. During this time social perceptions and attitudes towards safer sex may have changed. A more open social climate may have made it easier for students to talk about and cope with safer sex, and may have led to a greater willingness to accept knowledge. The present study also reported a significant increase in the proportion of participants who said they could refuse unprotected sex, and a large body of literature has reported that self-efficacy and willingness to use condoms among adolescents is strongly associated with actual condom use (30, 31). To promote the implementation of safer sex behaviours among VHS students, it is necessary to increase levels of knowledge about STIs/HIV prevention and self-efficacy to refuse unprotected sex.

Although condom use increased significantly, the difference in the number of sexual partners between the two surveys was not statistically significant. Considering that the number of 2 or more sexual partners accounted for about 1/3 of the total, only 35% of condom use in the second survey failed to prevent the transmission of STIs/HIV among students. No significant difference was found in self-reported experience of STIs diagnosis among study participants, suggesting that changes in sexual behaviour are not sufficient to reduce the risk of STI and that there is a need for STIs/HIV prevention services for VHS students. Students use condoms mainly for contraception rather than for STIs/HIV prevention (11, 32). Therefore, sex education and STIs/HIV prevention education in schools should help students improve their ability to protect themselves during sex, strengthen the awareness of the role of condoms in disease prevention, and eliminate the stigma against condoms and promote students' initiative to seek access to condoms.

The proportion of students reporting ever using condoms, ever using the pill, extracorporeal ejaculation and the safe period all increased by 15% or more, suggesting that there has been a significant improvement in students' awareness of the use of these types of contraception. While there has been a considerable improvement in contraceptive use among students, the actual use of all types of contraception remains low, indicating the need for enhanced contraceptive education. Given the range of contraceptive methods available to students, the fact that this study only looked at the proportion of students who had ever used contraception, the uncertainty surrounding the effectiveness of ejaculation and the safe period, and the fact that the choice of contraceptive method may be influenced by culture, personal preference and information received, the proportion of students who did not reduce their risk of pregnancies in this study requires further investigation. Future interventions should combine school-based education and community programmes. These interventions should develop gender-sensitive sexual health education modules that consider local social and cultural factors, with the aim of improving condom and other contraceptive uptake.

Our study reports a significantly higher percentage of oral sex among male VHS students, and the percentage in 2021 is significantly higher than the percentage of oral sex among 15–17 year old male adolescents in the United States (33), and even higher than the percentage of male students who have already had sexual intercourse in the 4th year of college in China (34). As society continues to open up, oral sex is gradually becoming more accepted in China. Yingying Huang reported that from 2000–2015, the proportion of Chinese men giving oral sex to women increased from 20.3%–36.8%, while the proportion of women giving oral sex to men increased from 16.2%–36.8% (35). While the risk of disease transmission from oral sex is much lower than from vaginal or anal sex, it can still transmit gonorrhoea, chlamydia, syphilis and HIV (36–38). Local and foreign adolescents rarely use protective measures during oral sex (27, 33, 34, 39), which may increase the risk of disease transmission through oral sex. Chinese studies have found that college students who engage in oral sex are at risk of having multiple sexual partners, non-regular sexual partners, sexual partners from the community or outside of school, less frequent condom use, and more frequent sex (33). The rapid increase in oral sex among VHS students may reflect the changing patterns of sexual behaviour among young people. Schools and health authorities should pay attention to this phenomenon and take targeted publicity and intervention measures.

Anal sex, whether homosexual or heterosexual, is a high-risk sexual behaviour involving a much higher risk of transmitting STIs/HIV than oral or vaginal sex. The significant increase in the rate of anal sex, coupled with the high proportion of multiple sex partners and the low rate of consistent condom use, means that the future risk of disease transmission through anal sex cannot be ignored. Surveillance of anal sex among VHS students should be strengthened in order to monitor the rate of anal sex and the protection level, and to provide data to inform targeted interventions.

This study has several limitations. The cross-sectional design of this study limits the ability to make causal inferences. Future research should adopt a prospective cohort study to gain a deeper understanding of the long-term causal effects of specific sex education strategies on students' acquisition of sexual health knowledge and adoption of healthy behaviours. The relatively small number of male students who reported having sex in the 2021 survey may limit the statistical power and generalisability of our findings. In addition, the self-reported nature of sexual behaviour may introduce bias due to social desirability or recall inaccuracies. Finally, the study was conducted in a single VHS, which may not be representative of all VHS students in Zhejiang Province or other regions of China. Therefore, our results should be interpreted with caution and further research with larger and more diverse samples is needed to confirm these findings.

## Conclusions

In conclusion, our study found that the proportion of male VHS students engaging in sexual behaviour decreased significantly, while their knowledge of safer sex, belief that they could refuse sex without a condom, consistent use of condoms, and ever use of condoms, the contraceptive pill, the safe period, and extracorporeal ejaculation for contraception increased. However, these positive changes are not significant enough to prevent STIs/HIV transmission and reduce pregnancy. This study reports a significant increase in the rate of oral and anal sex. Education and public health authorities can use these findings to inform prevention policies and create effective messages that respect cultural norms and address existing gaps. Firstly, schools should launch comprehensive sex education and STIs/HIV prevention programmes to raise students' awareness of condom use and contraception. Secondly, given the rising prevalence of non-vaginal sex, dedicated curricula should address misconceptions around STI/HIV transmission through these routes and promote harm reduction strategies. Thirdly, parent-child communication should be strengthened through family-focused initiatives that reinforce safer sex practices outside of schools. Finally, ongoing monitoring of sexual health trends among vocational students is critical in order to refine interventions and align them with evolving behavioural patterns. By adopting a multi-sectoral approach that combines education and community engagement, policymakers can effectively mitigate the transmission of STIs/HIV and unintended pregnancies in this vulnerable population.

## Data Availability

The datasets presented in this article are not readily available because The dataset we used involve sexual behaviour of young students which is sensitive in the context of China. Requests to access the datasets should be directed to Dr. Qiaoqin Ma, email:qqma@cdc.zj.cn.
